# Breath Analysis via Gas Chromatography–Mass Spectrometry (GC-MS) in Chronic Coronary Syndrome (CCS): A Proof-of-Concept Study

**DOI:** 10.3390/jcm13195857

**Published:** 2024-10-01

**Authors:** Marco Lombardi, Andrea Segreti, Marco Miglionico, Giorgio Pennazza, Lorenzo Tocca, Luca Amendola, Rocco Vergallo, Germano Di Sciascio, Italo Porto, Francesco Grigioni, Raffaele Antonelli Incalzi

**Affiliations:** 1 Department of Internal Medicine, University of Genova, 16132 Genova, Italy; mar.lombardi1993@gmail.com (M.L.); rocco.vergallo@gmail.com (R.V.); italo.porto@unige.it (I.P.); 2 Research Unit of Cardiovascular Science, Department of Medicine and Surgery, Università Campus Bio-Medico di Roma, 00128 Rome, Italy; m.miglionico@policlinicocampus.it (M.M.); g.disciascio@policlinicocampus.it (G.D.S.); f.grigioni@policlinicocampus.it (F.G.); 3 Cardiology Unit, Fondazione Policlinico Universitario Campus Bio-Medico, 00128 Rome, Italy; 4 Department of Movement, Human and Health Sciences, University of Rome “Foro Italico”, 00135 Rome, Italy; 5 Unit of Electronics for Sensor Systems, Department of Engineering, Università Campus Bio-Medico di Roma, 00128 Rome, Italy; g.pennazza@unicampus.it; 6 Dipartimento Prevenzione e Laboratorio Integrato, A.R.P.A. Lazio, 00173 Rome , Italy; lorenzo.tocca@arpalazio.it (L.T.); luca.amendola@arpalazio.it (L.A.); 7 Cardiothoracic and Vascular Department (DICATOV), IRCCS Ospedale Policlinico San Martino, Viale Benedetto XV, 6, 16132 Genova, Italy; 8 Unit of Internal Medicine, Fondazione Policlinico Universitario Campus Bio-Medico, 00128 Rome, Italy; r.antonelli@policlinicocampus.it

**Keywords:** chronic coronary syndromes (CCS), volatile organic compounds (VOCs), exhaled breath analysis, GC-MS, cardiac biomarkers

## Abstract

**Background:** This proof-of-concept study aimed to assess the diagnostic potential of gas chromatography–mass spectrometry (GC-MS) in profiling volatile organic compounds (VOCs) from exhaled breath as a diagnostic tool for the chronic coronary syndrome (CCS). **Methods:** Exhaled air was collected from patients undergoing invasive coronary angiography (ICA), with all samples obtained prior to ICA. Post hoc, patients were divided into groups based on coronary lesion severity and indications for revascularization. VOCs in the breath samples were analyzed using GC-MS. **Results:** This study included 23 patients, of whom 11 did not require myocardial revascularization and 12 did. GC-MS analysis successfully classified 10 of the 11 patients without the need for revascularization (sensitivity of 91%), and 7 of the 12 patients required revascularization (specificity 58%). In subgroup analysis, GC-MS demonstrated 100% sensitivity in identifying patients with significant coronary lesions requiring intervention when the cohort was divided into three groups. A total of 36 VOCs, including acetone, ethanol, and phenol, were identified as distinguishing markers between patient groups. **Conclusions:** Patients with CCS exhibited a unique fingerprint of exhaled breath, which was detectable with GC-MS. These findings suggest that GC-MS analysis could be a reliable and non-invasive diagnostic tool for CCS. Further studies with larger cohorts are necessary to validate these results and explore the potential integration of VOC analysis into clinical practice.

## 1. Introduction

Coronary artery disease (CAD) remains the leading cause of mortality, morbidity, and increased healthcare costs worldwide [1,2]. Currently, invasive coronary angiography (ICA) is the gold standard for diagnosing CAD. However, ICA is an invasive procedure with potential serious complications [3]. As such, identifying new cardiovascular risk factors and biomarkers associated with coronary plaque growth could help reduce reliance on invasive diagnostics [4].

Metabolomics is emerging as a powerful approach for identifying biomarkers linked to underlying metabolic disturbances in CAD [5]. Among the various metabolomic techniques, exhaled breath analysis has gained attention as an inexpensive, rapid, and non-invasive method for diagnosing and monitoring a range of diseases, including cardiovascular conditions [6]. Exhaled breath contains thousands of volatile organic compounds (VOCs) derived from both endogenous and exogenous sources, reflecting various metabolic processes in both health and disease [7]. Breath analysis offers a non-invasive way to study changes in VOCs, which are known to be clinically significant markers for several conditions, including heart failure [8,9,10].

Gas chromatography (GC) is a widely used technique for analyzing the qualitative composition of exhaled breath. It has been extensively applied to the study of various conditions, including lung cancer, asthma, chronic obstructive pulmonary disease (COPD), pneumonia, and COVID-19 infection [11,12]. This technique produces chromatograms that reveal the presence of various compounds in the sample based on retention times. When coupled with mass spectrometry (GC-MS), the identification of individual VOCs is even more precise and comprehensive. GC-MS is considered the gold standard for analyzing exhaled air, offering detailed insights into the molecular composition of VOCs [13,14].

Given the need for non-invasive and accurate diagnostic methods for CCS, we conducted a proof-of-concept study to evaluate the diagnostic and classificatory potential of GC-MS, comparing its performance to ICA, the current gold standard. Our study aimed to explore the breath profiles of individuals with CCS, identifying distinctive exhaled VOC patterns (breath fingerprints) that could potentially serve as non-invasive biomarkers for CCS diagnosis.

## 2. Materials and Methods

### 2.1. Study Population

Patients with suspected chronic coronary syndrome and a clinical indication for ICA (angina or equivalent, and/or positive evidence of ischemia in non-invasive tests, and/or presence of CAD in computed tomography angiography) who were admitted to the Cardiology Unit of the Campus Bio-Medico Hospital of Rome were enrolled. In line with the European Society of Cardiology guidelines, pressure guidewire-based functional assessments were carried out in patients with intermediate-grade stenoses on ICA to identify those causing ischemia. However, when severe stenoses were detected, revascularization was directly indicated without the need for further functional assessment [15,16]. The study included non-smokers or former smokers [17] aged 50 years or older in a clinically stable condition.

Exclusion criteria were established to avoid confounding factors that could affect breath analysis results. These included acute heart failure, valvular heart diseases requiring cardiac surgery, active smoking, asthma, neoplasms, renal failure (defined as a glomerular filtration rate < 60 mL/min/1.73 m^2^ based on the CKD-EPI equation), COPD, obstructive sleep apnea syndrome (OSAS), allergic rhinitis, atopy, an elevated total eosinophil count, recent respiratory tract infection or other respiratory diseases, excessive alcohol consumption, and inflammatory bowel diseases. Pulmonary function tests (PFTs) were performed the day before ICA, and patients exhibiting restrictive or obstructive respiratory patterns were excluded to ensure consistency in assessing the diagnostic potential of VOCs.

Post-ICA, patients were divided into two primary groups:

(a) Patients without an indication for myocardial revascularization;
(b) Patients with an indication for myocardial revascularization.

To further explore intra-class differences, a subgroup analysis divided the cohort into five subgroups:

- Group 1: patients without any lesion in the epicardial coronary arteries;
- Group 2: patients without significant lesions in the epicardial coronary arteries recommended for optimal medical therapy;
- Group 3: patients with lesions in small-caliber coronary arteries, recommended for optimal medical therapy;
- Group 4: patients with significant lesions in the epicardial coronary arteries, recommended for percutaneous coronary intervention (PCI);
- Group 5: patients with significant lesions in the epicardial coronary arteries, recommended for coronary artery bypass grafting (CABG).

### 2.2. Pulmonary and Cardiovascular Function Test

PFTs were performed in all subjects, including static lung volume assessments via the multiple-breath nitrogen washout technique (Quark PFT Cosmed), following the ATS/ERS Task Force guidelines on the standardization of respiratory function tests [18,19].

In addition, routine blood chemistry tests, epiaortic ultrasound scans, and transthoracic color Doppler echocardiograms (Philips IE33, Philips Medical Systems, Andover, MA, USA, and GE Vivid E9, GE Healthcare, Horten, Norway) were performed.

### 2.3. Exhaled Air Collection

Breath collection was performed between 07:00 and 10:00 a.m. prior to coronary angiography to minimize circadian variations in the metabolome that may affect the exhaled air composition [20]. Patients were instructed to fast and refrain from using toothpaste or consuming sweetened or alcoholic beverages before breath sample collection.

**Figure 1:**
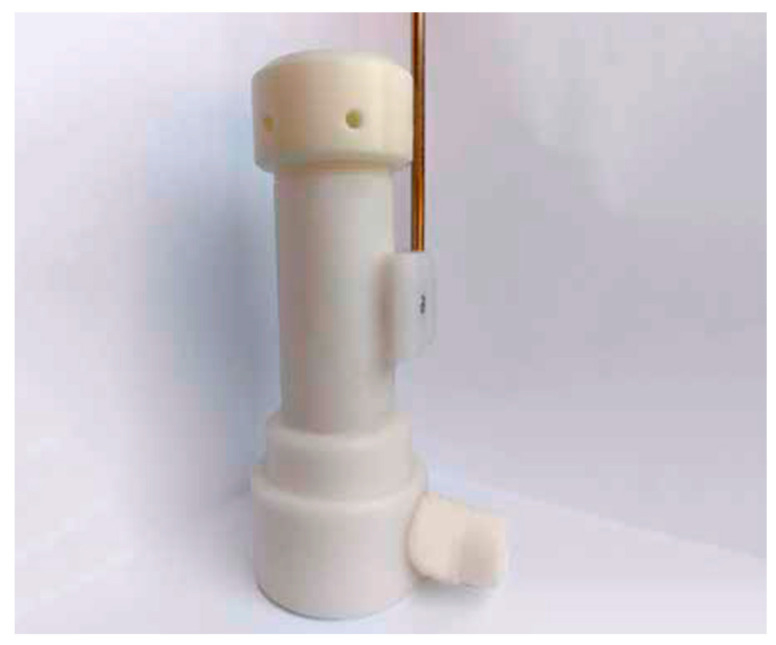
Exhaled air collection. Each patient provided a single sample by breathing tidally for three minutes into a dedicated storage device equipped with an adsorbing cartridge (Pneumopipe^®^). This device captures volatile organic compounds (VOCs) from the exhaled air for subsequent analysis using gas chromatography–mass spectrometry (GC-MS).

Each patient provided a single sample by breathing tidally for three minutes into a dedicated storage device equipped with an adsorbing cartridge (Pneumopipe^®^, European patent no. 12425057.2, Rome, Italy) (Figure 1). This sample was used for the gas sensor array evaluation, following previously described methods [21].

### 2.4. Analysis of Exhaled Air

#### 2.4.1. Analysis with Gas Sensor Array

Exhaled breath samples were collected on the adsorbent Tenax cartridges and processed through desorption into a sensor chamber. This was achieved using an interfacing device capable of heating the cartridge from 50 °C to 200 °C. The cartridge was subsequently cleaned by maintaining the temperature at 300 °C for five minutes.

#### 2.4.2. Analysis with GC-MS

To identify individual VOCs in the desorbed fractions from Tenax cartridges, the fractions were collected in 500 mL Tedlar bags [22] and analyzed using the carboxen solid phase microextraction (SPME) GC-MS technique [23]. GC-MS analysis was conducted with both the Total Ion Current (TIC) and Single Ion Monitoring (SIM) using an Agilent 5975 instrument [24].

Analytes absorbed on the SPME fiber were thermally desorbed in the GC injector at 280 °C. The chromatographic column had an initial temperature of 5 °C, maintained for 3 min with CO_2_. The temperature was then raised at a rate of 5 °C/min to 250 °C and held for 2 min. Data were derived from GC-MS chromatogram analysis, expressed as the ratio of the VOC area to the reference peak area.

The SPME technique allowed for the qualitative analysis of VOCs in exhaled breath. Additionally, an analytical study was conducted using activated carbon absorption tubes to enhance sensitivity and obtain quantitative information on the VOCs sought.

Breath samples were collected at Fondazione Policlinico Universitario Campus Bio-Medico in Rome, and the analysis was conducted at Agenzia Regionale per la Prevenzione Ambientale del Lazio (ARPA Lazio) in Rome.

**Figure 2:**
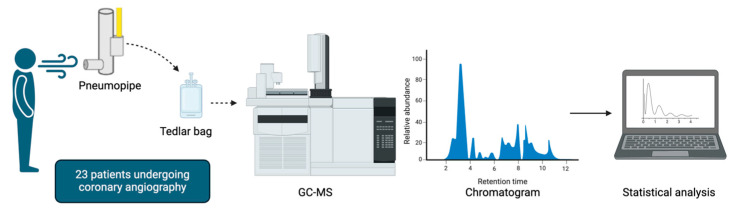
Exhaled breath collection and analysis. Each patient provided a single breath sample into a Pneumopipe^®^ device containing an adsorbing cartridge. Volatile organic compounds (VOCs) from Tenax cartridges were collected in 500 mL Tedlar bags and analyzed using the carboxen solid phase microextraction (SPME) gas chromatography–mass spectrometry (GC-MS) technique. The SPME fiber was thermally desorbed in the GC injector at 280 °C. Data were derived from GC-MS chromatogram analysis, expressed as the ratio of the VOC area to the reference peak area, and analyzed using a partial least square discriminant analysis (PLS-DA) model.

Figure 2 summarizes the exhaled breath collection and analysis performed in the present study.

### 2.5. Ethical Consent

The study was conducted in accordance with the Declaration of Helsinki [25] and was approved by the Ethics Committee of Campus Bio-Medico University of Rome (05/18 OSS—30/01/2018). Written informed consent was obtained from all participants.

### 2.6. Statistical Analysis

Continuous variables were expressed as means and 95% confidence intervals (CIs), while categorical variables were expressed as percentages. Statistical significance between means was determined using Student’s *t*-test or ANOVA, and Fisher’s exact test was used for categorical data.

A value of *p* < 0.05 was considered statistically significant. Statistical analyses were conducted using SPSS software, version 21.0 (SPSS Inc., Chicago, IL, USA).

A partial least square discriminant analysis (PLS-DA) model was performed on the 8-dimensional data array, which was cross-validated through leave-one-out cross-validation (LOOCV) to examine patients’ breath fingerprints before coronary angiography. PLS-DA was performed using the PLS Toolbox software (https://www.mdpi.com/1424-8220/21/13/4464, https://www.mdpi.com/2072-4292/13/20/4026, both accessed on 25 September 2024) (Eigenvector Research Inc., Wenatchee, WA, USA) in the MATLAB environment (The MathWorks Inc., Natick, MA, USA).

## 3. Results

**Figure 3:**
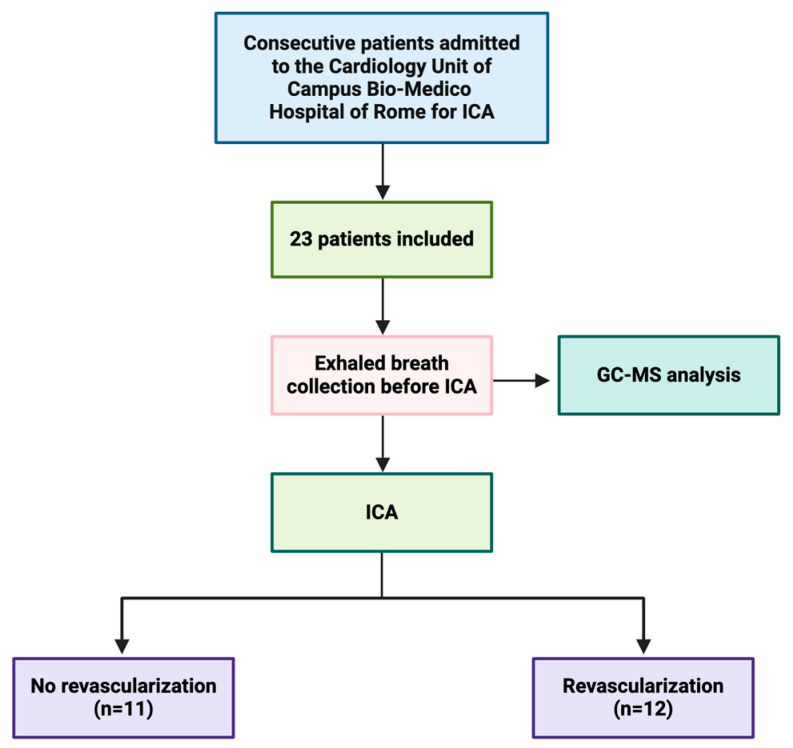
Experimental timeline of the study. Patients with suspected chronic coronary syndrome and a clinical indication for invasive coronary angiography (ICA) admitted to the Cardiology Unit of Campus Bio-Medico Hospital of Rome were enrolled in this study. A total of 23 patients were included. Exhaled breath collection and GC-MS analysis were performed before ICA. Based on the ICA findings, the population was divided into two groups: 11 subjects who did not require myocardial revascularization and 12 patients who required myocardial revascularization.

The study included a total of 23 patients, with 11 subjects not requiring myocardial revascularization and 12 patients requiring myocardial revascularization. Among the 11 patients in the non-revascularization group, ICA revealed either no coronary lesions, no significant coronary angiographic lesions, or lesions confined to small-caliber coronary arteries. Among the 12 patients requiring revascularization, some underwent PCI, while others underwent CABG. Figure 3 illustrates the experimental timeline of the study.

The anthropometric and demographic characteristics, as well as the mean values and significant comorbidities of the study population, are summarized in Table 1.

The mean age in both the revascularization and non-revascularization groups was over 65 years (67.09 ± 5.27 vs. 67.83 ± 4.5, *p* = 0.843), with an overall age range of 57 to 80 years. In the revascularization group, 92% of patients were men, whereas the non-revascularization group consisted of 55% men. The groups were similar in terms of body mass index (BMI), the prevalence of major comorbidities, smoking history, and the history of prior revascularization procedures (PCI or CABG). No significant differences in lung volumes or flows were observed between the groups. This similarity between the groups is an expected outcome given the study design.

When the population was divided into two groups based on the need for revascularization (Groups 1, 2, 3 versus Groups 4, 5), GC-MS correctly classified 10 out of 11 patients in the non-revascularization group (sensitivity of 91%) and 7 out of 12 patients in the revascularization group (specificity of 58%) (Table 2).

In the subgroup analysis, where the population was further divided into three categories comparing Group 1 versus Groups 2, 3, and Groups 4, 5, GC-MS accurately classified 1 out of 3 patients in Group 1, 4 out of 8 patients in Groups 2 and 3, and all 12 patients in Groups 4 and 5 (sensitivity of 100%) (Table 3).

**Figure 4:**
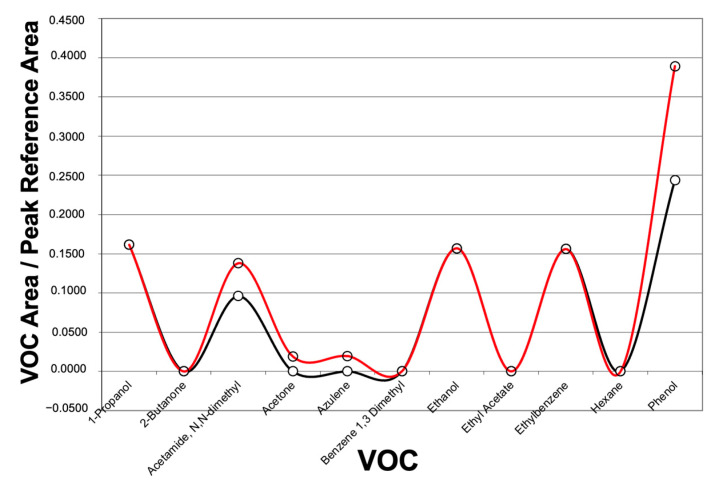
Graphical representation of data derived from gas chromatography–mass spectrometry (GC-MS) analysis. The results are expressed as the ratio of the VOCs’ area to the reference peak area on the y-axis, while the x-axis displays the VOCs detected in exhaled breath using GC-MS. The plot shows the distribution of VOCs from a patient included in the study who underwent revascularization. The red line represents the patient’s breath analysis, while the black line represents the control sample used for comparison.

Furthermore, 36 VOCs were identified through the GC-MS technique. The most frequently detected VOCs that allowed for discrimination between the patient groups and those associated with the diagnosis of CCS included the following: 1-propanol, acetamide, N,N-dimethyl, acetic acid, acetone, butane, ethanol, isopropyl alcohol, octane, 4-methyl-, pentane, 2-methyl-, phenol, and undecane (Table 4 and Figure 4).

These findings highlight the diagnostic potential of GC-MS in identifying breath profiles that distinguish between patients with and without the need for myocardial revascularization.

## 4. Discussion

In this study, we demonstrated that patients with CCS exhibit a unique breath fingerprint detectable by GC-MS. GC-MS showed high sensitivity (91%) in distinguishing between patients who did and did not require myocardial revascularization when grouped by clinical indications. Subgroup analysis further increased sensitivity to 100% when patients were categorized by the severity of coronary lesions. Rigorous patient selection minimized confounding factors that could affect measurements, ensuring that the results were unaffected by statistically significant differences in clinical features and comorbidities between the groups (all *p* > 0.05).

To our knowledge, this is the first study to evaluate the ability of GC-MS to detect breath fingerprints in CCS patients. Our results align with previous research using GC-MS in cardiovascular diseases, although most prior studies have focused on acute coronary syndrome (ACS). For instance, a previous study using plasma GC-MS demonstrated reproducible differences between ACS patients and controls [26]. Our study extends these findings to stable patients with CCS.

The use of non-invasive exhaled breath analysis could offer several clinical advantages over traditional diagnostic methods for CAD. Stress and exercise ECG tests, although widely used, have limited diagnostic accuracy in detecting coronary lesions, particularly in the early stages of CAD [27]. While imaging techniques like ICA or computed tomography are more accurate, they come with risks, such as radiation exposure and contraindications to stressors and contrast agents [3], and practical limitations, including the need for heart rate synchronization, challenges in patients with high calcium levels (especially in the elderly), and high costs [28]. In contrast, GC-MS-based breath analysis offers a non-invasive alternative that could lead to safer and more cost-effective methods for risk stratification in CCS patients.

In our study, GC-MS identified 36 VOCs (Table 4), including alkanes, alcohols, carboxylic acids, and ketones, which differentiated patient groups based on disease severity. These findings align with evidence that oxidative stress and inflammation—key processes in atherogenesis and plaque progression—can alter the chemical composition of exhaled air [7,29]. VOCs have been studied across various diseases [9,10,30,31,32,33], with those related to cholesterol metabolism, oxidative stress, bacterial metabolism in the intestine, and acetone metabolism being the most studied among cardiovascular diseases [6].

VOCs associated with lipid peroxidation, such as pentane and ethane, are produced during oxidative damage to cell membranes and have been found in higher concentrations in the breath of CAD patients [34,35,36]. Reactive oxygen species (ROS) play a key role in all stages of atherogenesis, contributing to plaque formation, progression, and rupture [37]. Additionally, Phillips and colleagues identified eight VOCs, including octane, 4-methyl- hexane, and pentane, which distinguish patients with unstable angina from healthy individuals, aligning with our findings in a stable setting [38]. Acetone, derived from the oxidation of 2-propanol, is another important VOC in cardiovascular diseases. Elevated acetone levels have been linked to heart failure, with studies showing correlations between exhaled acetone and heart failure severity, as measured by the New York Heart Association classification [9,39]. While several VOCs identified in the exhaled breath of CCS patients in this study have been previously associated with CAD, larger cohorts and more detailed pathophysiological studies are needed to validate these findings and further elucidate the mechanisms underlying VOC alterations in CAD patients.

While our study demonstrates the potential of GC-MS for CCS diagnosis, we also acknowledge the limitations of this method. Despite being considered the gold standard for breath analysis [13,14,40], GC-MS requires expert personnel, careful instrument calibration, and significant time for both sample processing and analysis. Samples must be transported from the clinic to the laboratory for analysis [40], and identifying low-concentration VOCs can be challenging, although storage in thermal desorption tubes allows samples to be preserved for extended periods. In contrast, our previous research with the electronic nose (BIONOTE-V) demonstrated promising diagnostic accuracy (78.3%) in identifying CAD patients requiring revascularization [41], suggesting that simpler, faster, and more practical alternatives could be developed for routine clinical use. While the current study shows that GC-MS provides superior sensitivity and reproducibility, the electronic nose may offer a practical option for clinical practice despite its limitations.

Overall, GC-MS remains highly sensitive, reproducible, and validated for analyzing VOCs in exhaled air, allowing for the creation of a “fingerprint” of various VOCs. Both GC-MS and the electronic nose hold promise for non-invasive CCS diagnosis, with the potential to significantly impact clinical management and the early detection of CAD.

## 5. Limitations

Several limitations of our study should be noted. Active smokers were excluded, as smoking can alter the breath pattern composition, although we did include former smokers and found no statistical differences in VOC profiles between those requiring revascularization and those who did not. Another limitation was that VOC analysis was performed on total exhaled air, which includes dead space air, typically for the first 125–150 mL of each expiration. We opted for a simple and reproducible method rather than more complex techniques to eliminate dead space air.

Other limitations include the lack of validation for our results and the exclusion of additional confounders inherent to the proof-of-concept nature of this study. We included patients with prior coronary revascularization to capture a broad range of CCS phenotypes [3], excluding only those with clinical presentations dominated by coronary artery thrombosis dominating the clinical presentation, such as acute coronary syndrome.

## 6. Conclusions

The results of this study suggest that patients with CCS exhibit a distinct VOC fingerprint in their exhaled breath. GC-MS has proven to be a precise and reliable method for characterizing these breath profiles in CCS patients. However, these findings require validation in larger cohorts with diverse comorbidities. Additionally, the diagnostic utility of this breath VOC fingerprint should be compared with other diagnostic methods, and the effects of PCI on breath VOC profiles should also be investigated.

Future research could focus on combining VOC analysis with other non-invasive diagnostic technologies to enhance the accuracy and early detection of CAD. Ongoing advancements in analytical techniques may make breath analysis more accessible and cost-effective for routine clinical use. If successfully integrated into clinical practice, breath analysis could serve as a non-invasive screening tool for at-risk populations, potentially enabling earlier interventions and personalized treatment strategies for managing cardiovascular disease.

## Figures and Tables

**Table 1:** Anthropometric characteristics and significant comorbidities in patients with an indication for revascularization and without.

	No Revascularization (*n* = 11)	Revascularization (*n* = 12)	*p* Value
Age	67.09 ± 5.27	67.83 ± 4.5	0.843
Male patients	54.54%	91.67%	0.06
BMI	28.76 ± 1.7	29.4 ± 2.4	0.714
Type 2 diabetes	18.18%	33.33%	0.640
Hypertension	100.00%	75%	0.210
Prior PCI/CABG	18.18%	25%	0.545
Smoking history	63.64%	75%	0.660
Dyslipidemia	72.78%	91.7%	0.316
Carotid atherosclerosis	72.78%	75%	0.640
EF	0.6 ± 0.04	0.56 ± 0.07	0.360
FEV1/FVC	78.6 ± 5.76	77.4 ± 4.47	0.744
SYNTAX score	3.3 ± 1.9	14.8 ± 8.4	<0.05

BMI = body mass index; EF = ejection fraction; PCI = percutaneous coronary intervention; CABG = coronary artery bypass graft; FEV1 = forced expiratory volume in the 1st second; and FVC = forced vital capacity.

**Table 2:** Confusion matrix of the partial least square discriminant analysis (PLS-DA) model among patients with and without the indication of myocardial revascularization.

Group 1, 2, 3 vs. 4, 5 GC-MS (*n* = 23)		
	Group 1, 2, 3	Group 4, 5
1, 2, 3	10	1
4, 5	5	7

**Table 3:** Confusion matrix of the partial least square discriminant analysis (PLS-DA) model in the subgroup analysis (Group 1 vs. Group 2, 3 vs. Group 4, 5).

	Group 1, 2, 3 vs. 4, 5 GC-MS (*n* = 23)		
	Group 1	Group 2, 3	Group 4, 5
1	1	1	1
2, 3	0	4	4
4, 5	0	0	12

**Table 4:** VOCs detected by gas chromatography–mass spectrometry (GC-MS). The reported VOCs in the table are derived from metabolism in the context of the dysregulation of lipids, amino acids, and carbohydrates. Specifically, they include alcohols, ketones, alkanes, aromatic hydrocarbons, acetic acid, and other compounds.

36 VOCs Detected by GC-MS		
1-Propanol	Dodecane	Isopropyl alcohol
2-Butanone	Dodecane + Decane,2,3,5,8-tetramethyl	Octane, 4-methyl
Acetamide, N, N, -dimethyl	Dodecane, 2,6,11-trimethyl	Pentane
Acetic Acid	Ethanol	Pentane, 2,3-dimethyl
Acetone	Ethyl acetate	Pentane, 2-methyl
Azulene	Ethylbenzene	Pentane, 2-mehtyl + cyclopentane
Benzene 1,3 dimethyl	Formic acid, butyl ester	1-Pentene
Butane	Hexane	Phenol
Butane, 2,3-dimethyl	Hexane, 2,3,5-trimethyl-	Styrene
Cyclopropane, ethyl	Hexane, 2-methyl	Trichloromethane
Decane, 2,4,6-trimethyl	Hexane, 3-methyl	Toluene
Decane, 3,7-dimethyl	Isobutane	Undecane

VOCs = volatile organic compounds; GC-MS = gas chromatography–mass spectrometry.

## Data Availability

Data from this study can be provided upon request to the corresponding author.
